# Nitrogen fixation in a non-heterocystous cyanobacterial mat from a mountain river

**DOI:** 10.1038/srep30920

**Published:** 2016-08-01

**Authors:** Esther Berrendero, Eduardo Fernández Valiente, Elvira Perona, Claudia L. Gómez, Virginia Loza, M. Ángeles Muñoz-Martín, Pilar Mateo

**Affiliations:** 1 Departamento de Biología, Facultad de Ciencias, Universidad Autónoma de Madrid, Darwin 2, 28049 Madrid, Spain; 2 Department of Botany, Faculty of Science, University of South Bohemia, 31 Branišovská, 370 05 České Budějovice, Czech Republic

## Abstract

*In situ* nitrogen fixation was investigated in a cyanobacterial mat growing on the bed of rocks of the Muga River, Spain. The filamentous non-heterocystous cyanobacterium *Schizothrix* dominated the mat, showing nitrogenase activity in the light at similar rates to those found in nearby heterocystous *Rivularia* colonies. N_2_ fixation in the light was significantly increased by an inhibitor of PSII and oxygen evolution, DCMU (3-[3,4-dichlorophenyl]-1,1-dimethylurea), and anaerobic conditions. However, no nitrogenase activity was found in the dark. Addition of fructose as a respiratory substrate induced nitrogenase activity in samples incubated under aerobic conditions in the dark but not in anaerobic conditions. Microelectrode oxygen profiles showed internal microaerobic microzones where nitrogen fixation might concentrate. Analyses of the 16S rRNA gene revealed only the presence of sequences belonging to filamentous non-heterocystous cyanobacteria. *nifH* gene diversity showed that the major phylotypes also belonged to this group. One of the three strains isolated from the *Schizothrix* mat was capable of fixing N_2_ and growing in the absence of combined N. This was consistent with the *nifH* gene analysis. These results suggest a relevant contribution of non-heterocystous cyanobacteria to nitrogen fixation in these mats.

Cyanobacteria are frequently key organisms in microbial mats in which the morphology, structure and colour are determined by the dominant species, the sediment features and other environmental factors. Several of the characteristic properties of this unique group of microorganisms, such as oxygenic photosynthesis and the ability of some cyanobacteria to fix atmospheric N_2_, have been proposed as important reasons why they are typically successful mat-building organisms^1^.

N_2_ fixation, an important process when mats grow in nutrient-depleted environments^2^, can be carried out by representatives of various bacterial taxa (called diazotrophs), and cyanobacteria are generally considered the principal diazotrophs in these ecosystems^3^,^4^,^5^. This process has often been studied in microbial mats from marine and hypersaline environments but has seldom been studied in rivers and streams^6^,^7^. Although substantial progress has been made towards understanding the ecological roles of cyanobacteria in freshwater benthic environments, little is known about their contribution to the N cycle via N_2_ fixation^8^.

We have been investigating various aspects of cyanobacterial communities in Spanish rivers, such as species composition and distribution as indicators of nutrient conditions^9^,^10^,^11^,^12^, the genetic and morphological characterization of strains^13^,^14^,^15^, and P metabolism^16^,^17^,^18^. In the Muga River, a small calcareous river in northeast Spain, we found a pink-pigmented cyanobacterial mat that dominated the bed of rocks of the river and consisted overwhelmingly of the filamentous non-heterocystous cyanobacterium *Schizothrix,* as previously recorded in this river^17^. The widespread distribution of this mat and the low level of combined nitrogen in the river led us to hypothesize that the *Schizothrix* mat could fix N_2_. Although cyanobacteria are globally abundant in freshwater benthic environments, the magnitude and importance of N_2_ fixation in lotic ecosystems is poorly understood^7^,^8^. Therefore, this study sought to investigate nitrogen fixation and potentially diazotrophic organisms in the *Schizothrix* mat as well as ecophysiological strategies related to the ability to perform nitrogen fixation in this habitat. *In situ* nitrogenase activity was measured, and sequences of 16S rRNA and *nifH* genes were examined to analyse diazotroph assemblages. Three cyanobacterial strains isolated from these mats were also characterized morphologically, genetically (16S rRNA and the *nifH* gene) and physiologically. Their ability to grow in a culture medium without combined N and to fix atmospheric N_2_ was also determined. To the best of our knowledge, this is the first study of N_2_ fixation in non-heterocystous cyanobacteria from running waters that is complemented by genetic information about these organisms.

## Results

### River environment

**Figure 1:**
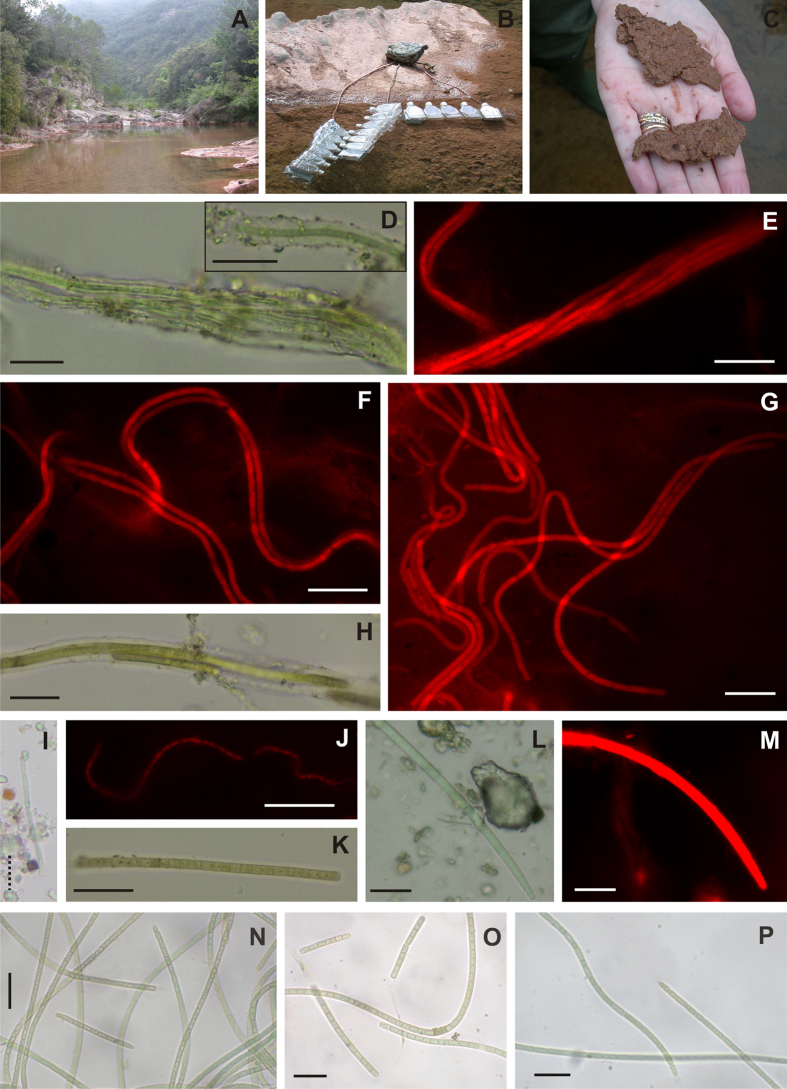
Photographs of the River Muga study site (**A,B**); photographs of the mat samples used in the *in situ* nitrogen fixation assays (**C**). Light and fluorescence microscopy photomicrographs showing the cyanobacterial morphotypes identified in the microbial mats. (**D–F**) *Schizothrix coriacea*, (**G,H**) *Schizothrix vaginatus*, (**I**,**J**) *Leptolyngbya* sp., (**K**) *Phormidium* sp. morphotype 1, (**L**,**M**) *Phormidium* sp. morphotype 2. Isolated strains from the microbial mats (**N**–**P**) MU50 (**N**) MU51 (**O**) MU52 (**P**). Scale solid bars, 20 μm; scale point bar, 8 μm.

The main physical and chemical characteristics of the water in the Muga River are that it is well oxygenated with an alkaline pH, a high calcium concentration, a low inorganic nitrogen concentration, and a low filterable phosphorus concentration (Table 1). Cyanobacterial communities are prevalent on its banks and in the riverbed, covering approximately 10% of the area. The most conspicuous and abundant cyanobacterium was a pink-coloured calcified mat of *Schizothrix,* which was situated on beds of rock in shallow areas along the banks (Fig. 1A–C). Other cyanobacteria included macroscopic *Rivularia* colonies that also grew in shallow areas with laminar flow. These colonies were used in this study as the reference heterocystous nitrogen-fixing cyanobacteria to enable comparison of the rates of nitrogenase activity (see below).

### Morphological study of the cyanobacterial composition of the *Schizothrix* mat

Light microscopy studies indicated that the microbial mat was dominated by two distinct morphotypes of *Schizothrix,* although other filamentous non-heterocystous cyanobacteria of the genera *Phormidium* sp. and *Leptolyngbya* sp. were also present, although at much lower frequencies (Fig. 1D–M). The most frequent morphotype was identified as *S. coriacea*, which showed long, densely intertwined filaments, typically with up to six trichomes, although it often only had one (Fig. 1D–F). The cells were 1.7–2.3 μm wide and the sheaths were firm, thin and non-lamellate, 4.4–9.1 μm wide, and usually had a colourless appearance under light microscopy. The second morphotype, which was classified as *S. vaginatus*, had wider trichomes (Fig. 1G–H) and was characterized by long, intertwined filaments that were enveloped by thick sheaths, usually colourless, 7–8 μm wide, and formed of cells 2.4–2.8 μm wide. The morphotype classified as *Leptolyngbya* sp. was characterized as having long trichomes, straight or flexuous and not attenuated at the ends, formed by cells up to 1 μm wide, and with apical rounded cells, without calyptra (Fig. 1I–J). Finally, two morphotypes of *Phormidium* sp. were observed. One exhibited straight trichomes with cells 3–3.5 μm wide, cross walls not narrowed, and a rounded end cell, without calyptra (Fig. 1K). The other morphotype had wider, 4.6–5.0 μm trichomes, which were slightly curved, gradually attenuated to varying extents towards the apex, with a rounded end cell and terminated by a calyptra (Fig. 1L–M). Heterocystous filaments comprised less than 0.1% of the biomass. Three cyanobacterial strains were isolated from these mats, which we designated strains MU50, MU51 and MU52. The morphology of these isolates was very similar, showing straight trichomes with unconstricted cross walls, cells 2.9–3.6 μm wide, and pointed conical or rounded end cells (Fig. 1N–P).

### Cyanobacterial 16S rRNA gene analysis

**Figure 2:**
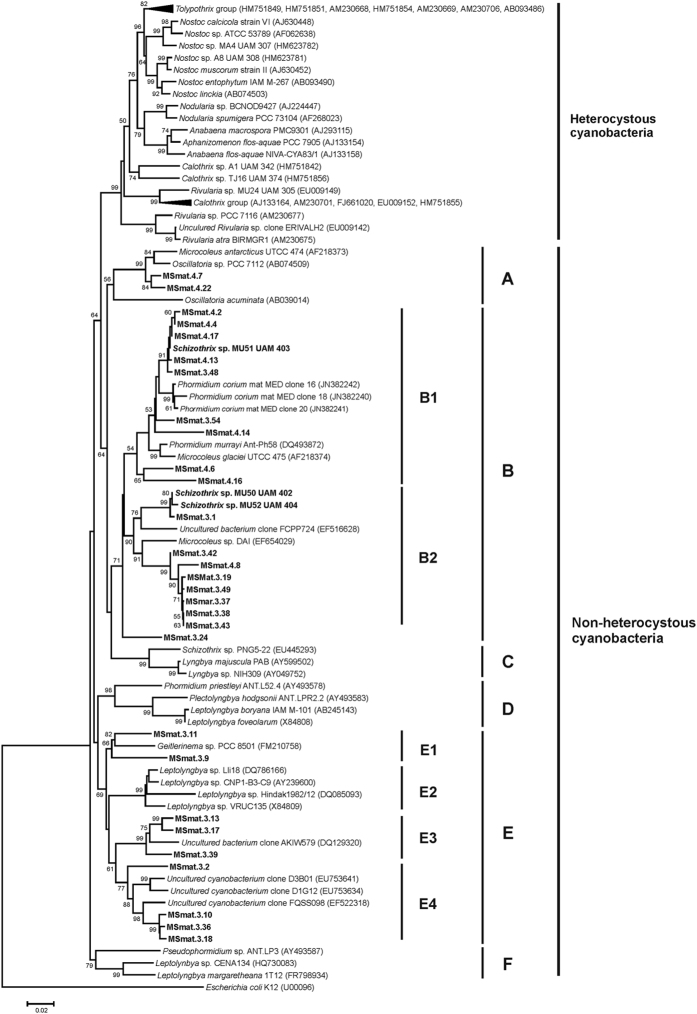
Phylogenetic tree based on 16S rRNA gene sequences (1382 positions) obtained by the neighbour-joining method. The sequences in the study recovered from the *Schizothrix* mat and isolated strains are indicated in bold. GenBank accession numbers for database sequences are in parentheses following the name. Bootstrap values (>50%) are noted above the nodes. The scale bar indicates 0.02 mutations per nucleotide position.

The genetic cyanobacterial diversity of the *Schizothrix* mat was examined by analysing the sequences of amplified cyanobacterial 16S rRNA genes. A total of 29 16S rRNA sequences were recovered in addition to the three isolates, all of which belonged to the order Oscillatoriales (Fig. 2). In the phylogenetic analyses, based on near-complete sequences of 16S rRNA, the sequences formed several clusters (A-E). The majority of the retrieved sequences were clustered in group B, which also included the sequences of the isolated strains. Strain MU51 was grouped with nine other sequences from the mats in subcluster B1. Subcluster B2 contained eight sequences from the mats and those corresponding to the strains MU50 and MU52. All other phylotypes were grouped in cluster E, which was further resolved into subclusters E1-E4. None of our sequences were included in the heterocystous cluster (Fig. 2). These results confirmed the microscopic observations highlighting the dominance of oscillatorian cyanobacteria in the *Schizothrix* mat.

### Physiological experiments with *Schizothrix* mats

Assays of nitrogenase activity (acetylene-reducing activity), photosynthesis (^13^C incorporation) and uptake of combined nitrogen (^15^NO_3_^−^ and ^15^NH_4_^+^ incorporation) were performed *in situ* (Fig. 1B) at midday in ambient light (1400 μmol photons m^−2^ s^−1^). To compare rates in this ecosystem, physiological activities were also measured in macroscopic colonies of heterocystous *Rivularia*, which were located close to the *Schizothrix* mats in the river^17^ (Table 2). The *Schizothrix* mat showed nitrogenase activity in the light at a similar rate to that of the *Rivularia* colonies, with no significant difference detected (t-test, P = 0.198) (Table 2). No nitrogenase activity was observed in either the *Schizothrix* mat or the *Rivularia* colonies incubated in the dark after 30 min of preincubation (data not shown). Specific photosynthetic activity and the uptake of combined nitrogen (^15^N-NO_3_^−^ and ^15^N-NH_4_^+^) were lower in the *Schizothrix* mat than in the *Rivularia* colonies (t-test: photosynthesis, P = 0.009; nitrate uptake, P = 0.026; ammonium uptake, P = 0.007) (Table 2). The *Schizothrix* mat had a low water content and a low ash-free dry weight/dry weight ratio, similar to those of the *Rivularia* colonies (Table 2). The chlorophyll (chl) content was significantly lower in the *Schizothrix* mat, whether referenced to dry weight or to ash-free dry weight (t-test, P < 0.001). Similarly, C content and, especially, N content were lower in the *Schizothrix* mat (t-test, P < 0.001). As a consequence of having a low N content, the *Schizothrix* mat had a higher C/N ratio than the *Rivularia* colonies (Table 2). Nevertheless, converting ethylene reduced the amount of nitrogen fixed, assuming the theoretical value of 3^19^ and comparing this value with those of the N-NO_3_^−^ and N-NH_4_^+^ incorporation in the *Schizothrix* mat and *Rivularia* colonies, we found that the contribution of N_2_ fixation to the total N incorporation in the *Schizothrix* mat (8.2%) was in the range of that of *Rivularia* colonies (4.2%), highlighting the importance of N_2_ fixation in the *Schizothrix* mat. The low physiological activity of the *Schizothrix* mat is in accordance with its low C and N content and suggests a slow growth rate for this mat.

**Figure 3:**
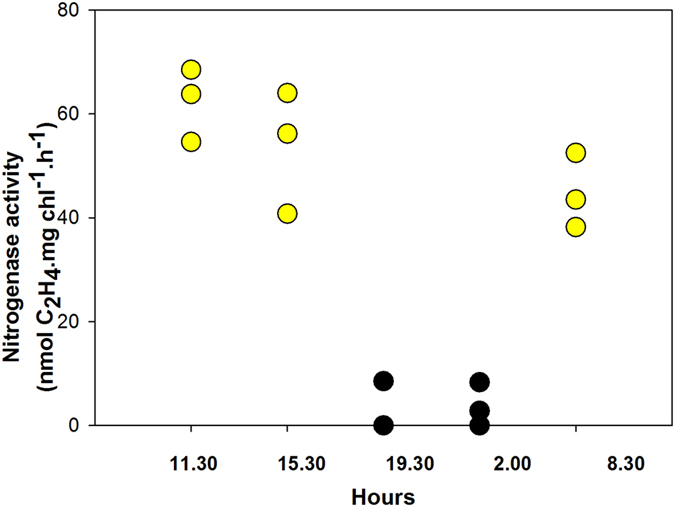
Diel cycle of nitrogenase activity in the *Schizothrix* mat from the Muga River. Assays were performed in the field under ambient light. Raw data are shown as scatter plots.

Figure 3 shows the diel cycle of nitrogenase activity in the *Schizothrix* mat. The cycle was subdivided into five periods: morning (9:30–13:30), afternoon (13:45–17:15), sunset (17:30–21:15), night (21:30–06:00) and sunrise (06:15–10:30). Nitrogenase activity was at its maximum at midday, decreasing thereafter, with very low values at sunset and at night, but increasing again in the morning. These results suggest a major role of light-dependent nitrogenase activity in this mat and therefore the importance of phototrophic cyanobacteria.

**Figure 4:**
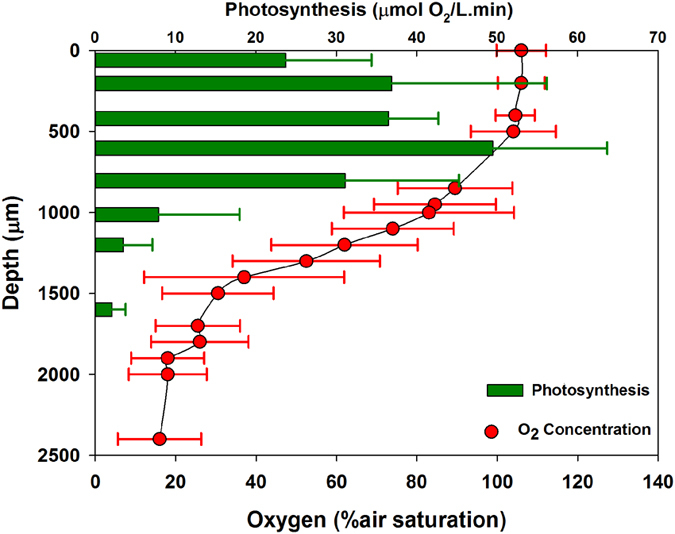
Vertical profiles of oxygen concentration and photosynthesis in the *Schizothrix* mat from the Muga River. Data (means and standard deviations) are derived from two profiles at different positions on each of the three replicates of each mat.

Vertical profiles of oxygen concentration and photosynthesis derived in the laboratory from mat samples under similar conditions to those in the field (Fig. 4) showed that oxygen concentration was more than 100% air saturation for the first 500 μm, followed by a sharp decline in concentration to 30% air saturation at 1500 μm. This decline was less pronounced thereafter, reaching 20% air saturation at maximum depth. Photosynthetic activity was maximal at approximately 500 μm and declined progressively to negligible values below 1700 μm (Fig. 4).

**Figure 5:**
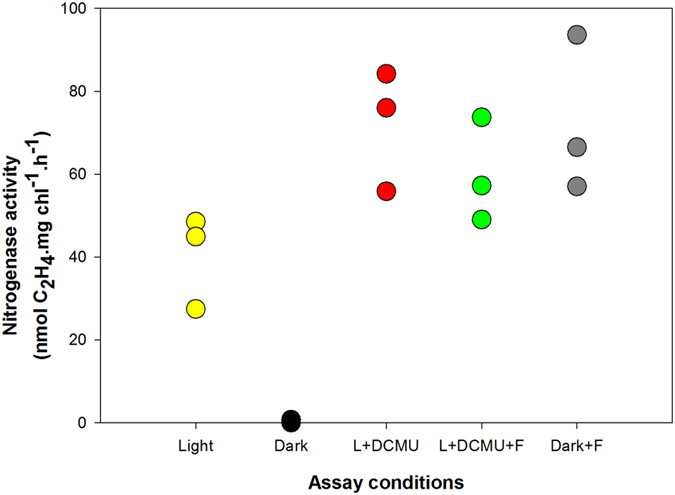
Nitrogenase activity in the *Schizothrix* mat from the Muga River assayed under different experimental conditions. Assays were performed in the field at midday under ambient light (1400 μmol photons m^−2^ s^−1^), or in the dark after 30 min pre-incubation in darkness. The chemicals were added 30 min before the start of the experiment. Raw data are shown as scatter plots.

The addition of DCMU, an inhibitor of PSII electron transfer and oxygen evolution, to the *Schizothrix* mat significantly increased nitrogenase activity in the light (t-test, P = 0.041) (Fig. 5), but the addition of the respiratory substrate fructose to the DCMU-supplemented mat did not induce a further increase in nitrogenase activity in the light. Again, no nitrogenase activity was found in the assays carried out in the dark, but the addition of fructose to the samples incubated in the dark induced substantial nitrogenase activity. These experiments were carried out at midday under the climatic conditions of the river with saturating light (>1000 μmol photons m^−2^ s^−1^) and saturated oxygen concentration in the water (100.6%).

The *Schizothrix* mat samples collected in the field were used to assess nitrogenase activity under anaerobic conditions in the laboratory because nitrogenase is inhibited by oxygen. Anaerobic conditions were obtained by gassing the samples with argon for 2 h. DCMU was added to the anaerobic samples incubated in the light to prevent photosynthetic O_2_ evolution. Incubation under anaerobic conditions did not induce nitrogenase activity in the dark even when fructose, as an exogenous carbon source, was added. However, the anaerobic conditions did induce a significant increase (t-test, P<0.001) in nitrogenase activity in the light (data not shown).

### *nifH* gene analysis

We retrieved 28 *nifH* sequences from *Schizothrix* mats, all of which shared similarity with cyanobacterial sequences reported in the GenBank database. Most of the sequences were tentatively classified as non-heterocystous Oscillatoriales-like *nifH* based on homology searches performed by comparing the sequences with the public protein databases. A group of nine sequences had similarity to sequences reported in the GenBank database as belonging to the heterocystous order Nostocales. The divergence of the *nifH* clones examined by DNA sequence ranged from 8.0–29.4%. It would appear that these cloned sequences represent novel populations because no complete similarity was found between our clones and the sequences in the database.

**Figure 6:**
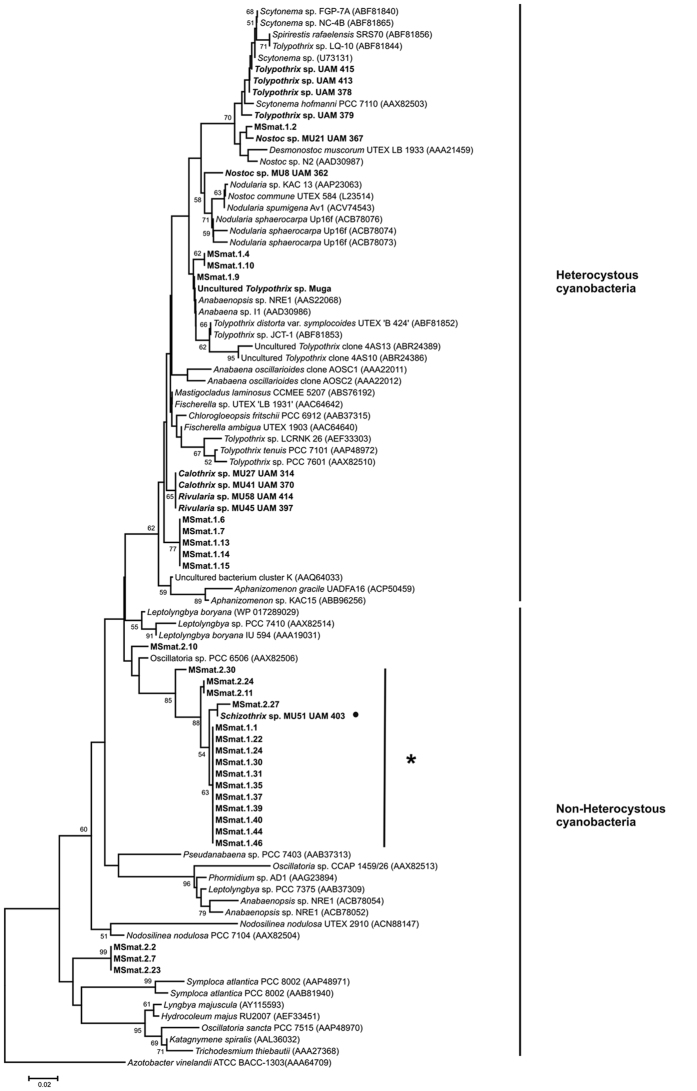
Neighbor-joining (NJ) phylogenetic tree inferred from nitrogenase amino acid sequences (sequences translated from a 374-bp internal fragment of *nifH*). GenBank accession numbers for database sequences are in parentheses following the name. Sequences obtained in this study are indicated in bold. The sequence from the isolated strain MU51 is indicated by a dot. Nucleotide sequences obtained from this study were compared with information available from the National Center for Biotechnology. Information database using the Basic Local Alignment Tool (BLAST) (http://www.ncbi.nlm.nih.gov/BLAST), and the most closely sequences related to the sequences obtained were downloaded where available. These sequences, our own ones, and that of an outgroup were used for the phylogenetic analysis. Bootstraps values >50% are indicated at the nodes. The scale bar represents 0.02 mutations per nucleotide positions.

Phylogenetic analysis enabled us to compare the *nifH* gene sequences obtained from the *Schizothrix* mat with those available in the public database representative of the orders Nostocales and Oscillatoriales. As most of the nitrogenase sequences published in the database belonged to uncultivated microorganisms that have not been identified, we also included sequences from the environmental samples and strains previously isolated from cyanobacterial communities in the Muga River (Fig. 6). This phylogenetic analysis highlights the clear separation of non-heterocystous and heterocystous cyanobacteria, in which the majority of *nifH* gene sequences recovered from the S*chizothrix* mat clustered with the sequences from the order Oscillatoriales. Most of these clustered with the sequence of the isolated strain MU51 (Fig. 6, asterisked). No PCR amplification of the *nifH* gene was noted for the two other isolates, MU50 and MU52. The Nostocales-like *nifH* sequences from the *Schizothrix* mat and the previously isolated heterocystous strains were distributed on different branches mixed with sequences of the database belonging to order Nostocales.

### Physiological experiments with isolated strains

**Figure 7:**
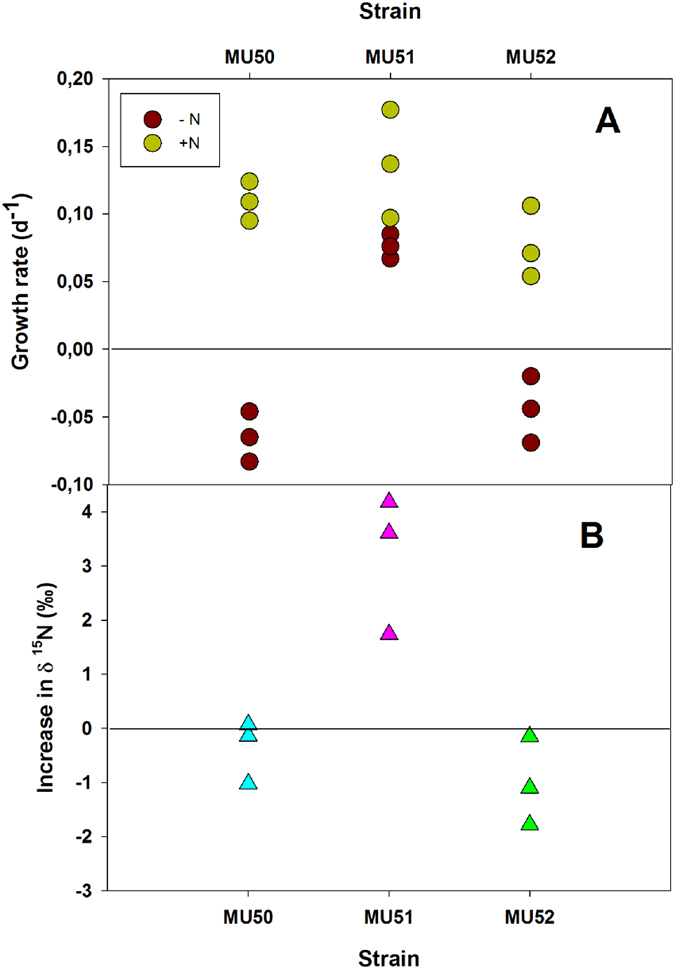
Growth rate (**A**) and increase in δ^15^N with respect to natural abundance (**B**) in three strains isolated from the *Schizothrix* mat. Cells were grown for two weeks in a medium with nitrate (green boxes) or in a medium without combined nitrogen (brown boxes). The incorporation of ^15^N_2_ was determined in cells cultured for 24 h in a medium without combined nitrogen under aerobic conditions in air supplemented with 10% ^15^N_2_ (99% enrichment). The raw data are shown as scatter plots.

The three isolated strains had low growth rates when grown without gassing or stirring on a nitrate medium (Fig. 7A). There were no significant differences between the strains (ANOVA, P = 0.378). All three had a generation time of approximately 9 days. Only the MU51 strain was able to grow in a medium without combined N and maintained its characteristic green colour throughout the experiment. Growth rates were slightly lower than on a medium with nitrate (generation time, 13 days), although the differences were not significant (t-test, P = 0.273). This result suggests that MU51 was able to fix atmospheric N_2_ and grow with N_2_ as the only source of nitrogen. The other two strains did not grow on the medium without combined nitrogen. They exhibited a progressive decline in chlorophyll concentration until they were pale yellow in colour at the end of the experiment.

The low growth rates of these strains made it difficult to obtain sufficient biomass to assess their ability to fix N_2_ with the acetylene reduction assay. Therefore, to avoid a long incubation with acetylene, which can be toxic for the cells^20^, we used the most direct and accurate method of incorporation of ^15^N_2_.

Consistent with the findings presented above and confirming its ability to fix N_2_, the MU51 strain showed a small but significant increase in δ ^15^N with respect to natural abundance (t-test, P = 0.038) when the cultures were maintained under aerobic conditions for 24 h with 10% ^15^N_2_ (99% enrichment) (Fig. 7B). The other two strains did not show any increase in δ ^15^N, indicating that they were not able to incorporate ^15^N_2_. This was confirmed by their lack of growth in the absence of combined nitrogen.

## Discussion

### Non-heterocystous cyanobacteria as dominant organisms in the microbial mats

Nutrient loading, concentration and ratios are strong selective forces shaping cyanobacterial communities in running waters. Large increases in the proportion of N_2_-fixing cyanobacteria in periphyton communities have been found in response to low concentrations of dissolved inorganic nitrogen (DIN) in river water, especially N-NO_3_^−^ and N-NH_4_^+^^9^,^12^,^21^,^22^,^23^. Similar to other mountain rivers, the Muga River showed a low level of combined N. However, the dominant community is a pink-pigmented cyanobacterial mat (see Fig. 1A,B) for which microscopic examination showed it to be overwhelmingly composed of non-heterocystous cyanobacteria identified morphologically as belonging to the genus *Schizothrix*, as previously recorded in this river^17^. This finding is consistent with that of Stal^1^, who reported that the vast majority of microbial mats are composed of non-heterocystous cyanobacteria. However, the *Schizothrix* mat in this river has a simple structure because it develops directly on the bed of rocks and lacks the deep layers of sediment characteristic of most microbial mats. This simplicity is reflected in the 16S rRNA gene diversity, the analysis of which revealed only the presence of sequences belonging to filamentous non-heterocystous cyanobacteria (Oscillatoriales). The sequences of our *Schizothrix* cultures as well as the majority of the other cloned sequences from this mat were included in the same cluster. Another *Schizothrix* sequence from the database, which was also included in the tree, is not closely related to the sequences obtained from the mat, most likely related to its occurrence in a different (marine) habitat^24^. Similar results have been found in freshwater benthic mat-forming cyanobacteria isolated from New Zealand^25^ and other environments, including extreme polar environments and thermal springs^26^,^27^, whereby filamentous types of cyanobacteria from the order Oscillatoriales with thin trichomes were found to be the most abundant group. It has been argued that the absence of heterocystous cyanobacteria from the microbial mats is due to their lack of gliding motility, which is mainly a characteristic of non-heterocystous cyanobacteria^1^. This property is important for optimal vertical positioning and allows for the compensation of the rapidly shifting physicochemical gradients in microbial mats^1^.

The fact that non-heterocystous cyanobacteria were dominant in a river with low levels of combined nitrogen is striking because to the best of our knowledge, no diazotrophic abilities have been described for non-heterocystous cyanobacteria from running waters. Given its potential for nitrogen fixation and similar values in nitrogenase activity to those found in nearby heterocystous *Rivularia* colonies, other specific traits may explain niche differentiation. Many mat-forming *Schizothrix* populations grow in aquatic environments that are subject to alternating periods of great moisture or even being submerged with periods when they are almost or completely dry^28^. *Schizothrix* mats from the Muga River typically grow in shallow areas with only a thin film of water under low flow and are conspicuous on the dry riverbank. The brick colour typical of the outer part of the mats exposed to full daylight^28^ was observed in these parts of the river (see Fig. 1A), most likely as a result of the UV-protection of the extracellular red pigment gloeocapsin in the sheath of trichomes^29^. Therefore, in addition to potential nitrogen fixation, specific ecological niche occupancy in a system with changing flows, such as the Muga River at this site^17^, may explain the dominance of *Schizothrix* mats in these running waters. Cantonati *et al*.^30^ found clear patterns in the depth distribution of benthic cyanobacteria and algal pigments that indicated adaptation to environmental pressures such as water level fluctuation and light attenuation.

### Nitrogen fixation and diazotrophic organisms in the *Schizothrix* mat

The results show that the *Schizothrix* mat fixes N_2_ in the light at similar rates to those of a heterocystous cyanobacterium located nearby in the river. It should be noted that these rates are lower than those reported for other cyanobacterial mats, such as those from marine habitats^31^. However, rates of nitrogenase activity in running waters from mountain ecosystems or similar systems with low water temperatures are typically low^32^. The results of our N_2_ fixation survey were similar to those obtained from subalpine oligotrophic streams in the Rocky Mountains of North America^33^,^34^ and from Antarctic streams^35^,^36^. A few exceptions have shown that N_2_ fixation rates in streams can be higher. Grimm and Petrone^31^ reported high rates in desert streams during summer and autumn. Kunza and Hall^37^ found that average N_2_ fixation rates were low in nine out of twelve streams in Grand Teton National Park, Wyoming. Variations in N_2_ fixation rates have been explained in terms of the availability of inorganic N but also with respect to temperature and light^31^,^33^,^34^,^37^. Nitrogenase activity is greater at lower NO_3_^−^ concentrations in the stream waters, although this relationship was shown to be nonlinear^34^,^37^. Nonetheless, it should also be noted that N_2_ fixation rarely exceeds nitrate or ammonium uptake^37^. Marcarelli and coworkers^7^ compared N_2_ fixation rates from a wide variety of streams, reporting that the median N fixation was 80–130 times lower than median DIN uptake fluxes. They highlighted the fact that in-stream N_2_ fixation rarely contributes 5% of the annual N input to N budgets. This is a similar value to those that we have found. On the other hand, ambient stream water temperature directly affects N_2_ fixation. Increased temperatures stimulate N_2_ fixation in all streams analysed^31^,^32^,^33^,^37^. However, it is difficult to discern which factor exerts the strongest influence because these parameters tend to covary^31^. Further research is needed to quantify the incidence of such characteristics, which may help explain the spatial and temporal variation in N_2_ fixation rates.

The finding that nitrogenase activity occurs in the light was unexpected because it has been widely reported that cyanobacterial mats that are dominated by non-heterocystous cyanobacteria usually only show substantial rates of nitrogenase activity in the dark^38^,^39^. These results raised the question about what kind of diazotrophs are responsible for the N_2_ fixation in this mat. The addition of DCMU to the *Schizothrix* mat produced a significant increase in nitrogenase activity, as usually occurs in non-heterocystous cyanobacteria^1^,^3^,^39^, whereas the addition of DCMU to heterocystous cyanobacteria caused a significant decrease in nitrogenase activity in the light^1^,^4^,^40^. The results of the laboratory experiments also indicated that at least one of the main strains of non-heterocystous cyanobacteria from the *Schizothrix* mat can fix N_2_. This is also consistent with the results of the analysis of the *nifH* gene. The majority of *nifH* sequences retrieved from the samples were related to Oscillatoriales-type *nifH*. Some of these sequences belonged to one of the dominant *Schizothrix* morphotypes isolated, which in turn was able to grow in the absence of combined nitrogen and to exhibit nitrogenase activity. These results suggested that nitrogen fixation in these mats may be attributed to non-heterocystous cyanobacteria. However, it has been argued that different mechanisms such as *nifH*-degenerated primers, polyploidy in cyanobacteria and differential DNA extraction could bias *nifH* clone libraries against heterotrophic diazotrophs^41^. Therefore, the importance of these nitrogen-fixing organisms may have been underestimated in analyses of early successional biological soil crust communities^41^. Therefore, although our results indicate that the primary diazotrophs in the mats are cyanobacteria because all the recovered sequences belonged to this group, in spite of the use of general primers for aerobic and microaerophilic diazotrophs, we cannot discount the potential for fixation by non-phototrophic bacteria stimulated by cyanobacteria-released metabolites in the light^42^.

### Ecophysiological strategies

The ability to fix nitrogen in the light requires the development of mechanisms for protecting the O_2_-labile nitrogenase^38^. Paerl and coworkers^43^,^44^ emphasized the importance of the formation of anoxic microzones in microbial mats and other systems in which N_2_ fixation could proceed in an uninhibited manner. In our study, the results from oxygen microelectrodes indicate the existence of internal O_2_-reduced microzones where nitrogen fixation may be concentrated. The saturated levels of oxygen found in the upper layers of the photosynthetically active mat would most likely restrict nitrogenase activity to the deeper layers (1300–1600 μm in depth) where the O_2_ concentration is well below saturation. Stal *et al*.^45^ found the highest nitrogenase activity in the lowest layer of a cyanobacterial mat (2–3 mm), indicating a spatial separation of N_2_ fixation (lower layers) and oxygenic photosynthesis (top layer).

In addition, we propose that enhancement of oxygen-scavenging mechanisms, such as high respiration rates, may be supplementary ways of protecting nitrogenase activity, as has also been proposed for *Trichodesmium*^43^. The addition of DCMU had an alleviating effect due to the decreased O_2_ production at the water-splitting site of PSII, thereby allowing nitrogenase activity to attain its maximal rate. Under these conditions, ATP had to be supplied mainly by cyclic phosphorylation and the reductant supplied by aerobic respiration, which in turn decreased O_2_ concentration. Under strictly anaerobic conditions in the light, nitrogenase also showed maximal rates of activity. Here, again, ATP had to be supplied by cyclic phosphorylation, whereas the reducing power could be derived from fermentative metabolism. Fermentative NADH most likely feeds electrons to the nitrogenase enzyme through PSI, as was reported in isolated heterocysts by Schrautemeier and Böhme^46^. Dark aerobic conditions did not facilitate significant levels of nitrogenase activity in the *Schizothrix* mat unless exogenous fructose was supplied. The addition of fructose increased respiratory metabolism, thereby decreasing oxygen tension and increasing the supply of ATP and reductant to a level compatible with maximal rates of N_2_ fixation. However, under anaerobic conditions, exogenous fructose failed to support nitrogenase activity, indicating that fermentative metabolism by itself cannot provide enough ATP and reductant for nitrogen fixation.

The Mehler reaction, which occurs in cyanobacteria via direct photoreduction of O_2_ to water (without the production of significant amounts of reactive oxygen species, as occurs in eukaryotes^47^), may also alleviate the negative effect of increasing O_2_ production at PSII. In addition, increased Mehler activity could generate excess ATP through cyclic electron transport, which is needed to support nitrogen fixation^48^,^49^,^50^.

In conclusion, although we cannot discount the possibility that non-phototrophs contribute to nitrogen fixation in the *Schizothrix* mat, all of our results (the microscopic observations, the 16S rRNA and *nifH* gene analyses, the effect of DCMU on nitrogenase activity, and the physiological experiments on isolated strains) are consistent with our hypothesis that non-heterocystous cyanobacteria are driving nitrogen fixation in the mats. These diazotrophs may be important contributors to the nitrogen fixed in this river and merit further scrutiny.

## Methods

### Study site and sampling

Field studies were carried out in the Muga River, Girona, northeast Spain, at a site 10 km downstream from its source in the Pyrenees (42°22′ N, 2°42′ W; UTM ED50 47 48 24 4684320; 310 m.a.s.l.). The field site and water sampling and analysis procedures have previously been described in detail^17^. Samples of the cyanobacterial communities were collected with the help of a knife blade because their structure does not allow the use of a metal core. The fresh weight of all the samples was determined with a field balance after gently placing the samples on filter paper to eliminate water droplets. Samples for determinations and genetic analysis were frozen in liquid nitrogen before transportation. The samples used to measure nitrogenase activity in the laboratory were kept in darkness at 4 °C until further use. Air irradiance during the experiments was measured every 10 min using a Li-Cor LI-1000 data logger equipped with a 2π quantum sensor. Fieldwork was carried out from 9 to 11 May 2009. The river flow during this time was moderate (average of 0.16 m s^−1^).

### Community and biomass analyses

Morphological observations of microbial mats were made under a dissecting microscope (Leica, Leica Microsystems) and an Olympus BH2-RFCA photomicroscope equipped with phase-contrast and epifluorescence. Morphometric measurements were made from digital images captured with an Olympus DP 20 digital camera (high resolution; 1600 × 1200 pixels) interfaced with an Olympus CX41 microscope. Morphological identification followed the taxonomic keys of Komárek and Anagnostidis^51^ and Whitton^28^. All of the important key features that were taken into consideration to identify natural samples, such as cell width, cell length, attenuation of apical cells, and sheath morphology, were studied in samples of 25–50 cells. Dry weight was measured by weighing the samples after drying at 60 °C for 24 h. The ash-free dry weight was measured by weighing the dried samples after mineralization at 450 °C for 2 h and calculating the difference between the dry weight and the weight after mineralization. Chlorophyll *a* (Chl *a*) content was determined by overnight extraction with 90% acetone using samples that were previously sonicated for 10 min in the dark. After centrifugation at 9,600 *g*, absorbance was measured at 665 nm using a Hitachi U2000 spectrophotometer. This procedure was repeated with the same sample until no more pigment could be extracted. To determine the C and N content, the samples were dried at 60 °C for 24 h, ground by mortar and pestle and analysed using an elemental analyser (LECO CHNS-932) with a thermal conductivity detector. The percentage abundance in natural samples was evaluated by counting the presence of each morphotype (as cells in a filament or as equal numbers of individual cells).

### Cyanobacterial isolation

To simulate the chemical conditions of the river water, cyanobacteria were isolated on Petri dishes with a 1.5%-modified CHU No 10^52^ agar medium in which P was reduced to 1.5 mg l^−1^ and calcium was increased to a final concentration of 0.15 g l^−1^. Three strains were successfully isolated from the *Schizothrix* mats from the Muga River, each from a single trichome. The strains have been deposited in the culture collection of the Universidad Autónoma de Madrid (UAM), Spain, designated with the reference numbers MU50 UAM 402, MU51 UAM 403 and MU52 UAM 404. The isolated strains were maintained under light:dark periods of 16:8 h at 18 °C. The light intensity during the light period was 20 μmol photon m^−2^ s^−1^.

### Photosynthetic activity

Photosynthetic activity was measured using the stable isotope technique (^13^C uptake) as previously described^53^. To prepare the assay, 10% of the natural dissolved inorganic carbon (DIC) concentration of the water was added as H^13^CO_3_ (99% ^13^C atoms) (Cambridge Isotope Laboratories, MA, USA). ^13^C isotopic enrichment was measured with an IRMS Micromass-Isochrom mass spectrometer. The DIC content of the water was calculated from its alkalinity, determined by acid titration, taking the pH and temperature into consideration.

### Microelectrode studies of light-saturated photosynthesis

Gross oxygenic photosynthesis was estimated at depth intervals of 200 μm through the vertical profile of the microbial mat samples with a polarographic Clark-type oxygen microelectrode (diameter 50 μm) (Diamond General, Ann Arbor, MI, USA) as previously described^36^. Two profiles of photosynthetic activity were derived at different positions on each of the three replicates of each mat.

### Uptake of ammonium and nitrate

The uptake of N from ammonium and nitrate was measured using the stable isotope technique as previously described^53^. To prepare the assay, 10% of the natural water concentration of N-NH_4_^+^ and N-NO_3_^−^ was added as (^15^NH_4_)_2_SO_4_ (98.0% of ^15^N atoms) or as K^15^NO_3_ (99.9% of ^15^N atoms) depending on the experiment. ^15^N isotopic enrichment was measured using an IRMS Micromass-Isochrom mass spectrometer (model Thermo Delta V Advantage) coupled with a high-temperature Thermo Flash 1112 Elemental Analyzer.

### Nitrogenase activity

Nitrogenase activity of the *Schizothrix* mats was measured using the acetylene reduction technique as previously described^4^. In the field experiments, samples of the mats were incubated *in situ* in triplicate in 75 ml flat plastic bottles (1 g fresh weight per bottle) containing 15 ml filtered river water. Control bottles containing river water without mat samples were also incubated to check for other possible sources of ethylene aside from the cyanobacterial communities. The plastic bottles were capped with reversible rubber stoppers and covered with parafilm to ensure gas-tightness. A total of 10% of the air inside the bottles was replaced by the same volume of chemically pure acetylene (Carburos Metalicos, Madrid, Spain), which contained approximately 0.05–0.075 nmol/ml of contaminant ethylene. The samples were incubated for 4 h on the streambed to maintain natural conditions of temperature and illumination (see Fig. 1B). The samples were secured to the streambed as described above. Special care was taken to ensure that the samples were positioned with the surface layer facing upwards and to avoid overlapping. At the start and at the end of incubation, duplicate gas samples were collected with double needles in 10 ml pre-evacuated BD Vacutainer tubes (Belliver Industrial Estate, Plymouth, UK). These tubes give a peak at the same retention time as a concentration of ethylene equivalent to 0.05 nmol/ml. The ethylene concentration was determined in 1 ml gas samples using a gas chromatograph (Shimadzu model GC-8A) equipped with a flame ionization detector and Porapak N 80/100 column. The detection limit of the chromatographic method was 0.01 nmol ethylene/ml. To improve the reliability of the data, any values below 0.05 nmol ethylene/ml were not taken into consideration. To eliminate any errors arising from contaminants, ethylene production was quantified as the difference between the final and initial samples. A mat sample was considered to exhibit nitrogenase activity if differences in ethylene concentration between the initial and final gas samples were significant (paired t-test). Samples incubated in the dark were covered with aluminium foil and kept in darkness at ambient water temperature for 30 min before adding the acetylene. Fructose (1 mM) and/or DCMU (3-[3,4-dichlorophenyl] - 1,1 dimethylurea) (10^−5^ M) were added 15 min before adding the acetylene.

In the laboratory experiments, samples of the *Schizothrix* mat were incubated in triplicate in 40-ml Erlenmeyer flasks (approximately 0.5 g fresh weight per flask) containing 10 ml of filtered river water. Samples were incubated at room temperature (22 °C) with a constant saturating illumination of 450 μmol photons m^−2^ s^−1^. Anaerobic conditions were obtained by gassing the samples with argon for 30 min. DCMU (10^−5^ M) was added to those anaerobic samples incubated in the light to prevent photosynthetic oxygen evolution. Before starting the experiment, samples were kept in filtered river water at room temperature for 20 h to allow full rehydration and acclimation to room temperature. DCMU or fructose (1 mM) was added at the same time as the filtered water. Gas samples of 1 ml were taken at the start and finish of the incubation period, and ethylene production was measured as described above.

Nitrogen fixation of the isolated strains was measured using the stable isotope ^15^N_2_. The samples were incubated in 10 ml Vacutainer tubes, which were filled to overflowing with culture medium without nitrogen before being carefully sealed with a septum cap. A gas-tight syringe was used to inject 0.5 ml of ^15^N_2_ (99% of ^15^N_2_ atoms) (Cambridge Isotope Laboratories, MA, USA) into each tube. Finally, a 0.5-ml volume of the solution was withdrawn to equalize the pressure across the septum. Two replicates plus one control (without ^15^N_2_ gas) were incubated with light:dark periods of 16:8 h at a temperature of 18 °C and a light intensity of 20 μmol photons m^−2^ s^−1^ during the light period. After incubation, samples were rinsed three times with culture medium without nitrogen to eliminate any unincorporated ^15^N. The samples were dried at 60 °C and ground with a mortar and pestle. Additional samples of each culture were taken before the start of the experiment to determine the natural abundance of ^15^N. The natural abundance of ^15^N and isotopic enrichment were measured using an IRMS Micromass-Isochrom Mass Spectrometer.

### Genetic analyses

DNA was extracted as previously described^11^,^21^. The *nifH* segment (374 bp) was amplified using two primer sets designed by Olson *et al*.^54^. One of the primer sets was designed to amplify aerobic and microaerophilic diazotrophs (forward primer: 5′-ATXGTCGGXTGXGAXCCYAARGC- 3′; reverse primer: 5′-ATGGTGTTGGCGGCRTAZAKYGCCATCAT- 3′, where X  =  C or T, Y  =  G or C, R  =  G or A, Z  =  C, G, or A, and K  =  G or T), while the other preferentially amplified cyanobacterial *nifH* fragments (forward primer: 5′-CGTAGGTTGCGACCCTAAGGCTGA-3′; reverse primer: 5′-GCATACATCGCCATCATTTCACC-3′). The PCR conditions are described in Díez *et al*.^55^ and Zehr *et al*.^56^. Almost the entire 16S rRNA gene, the intergenic transcribed spacers, and part of the 23S rRNA gene (approximately 1800 bp) were also amplified from the DNA using the universal 16S ribosomal DNA primer pA (forward primer: 5′-AGAGTTTGATCCTGGCTCAG-3′)^57^ and cyanobacteria-specific B23S (reverse primer: 5′-CTTCGCCTCTGTGTGCCTAGGT-3′)^58^ under previously described conditions^59^. The amplified products were separated by cloning using a Qiagen PCR Cloningplus kit following the manufacturer’s instructions. Recombinant clones carrying the correct size insert were purified and sequenced as previously described^11^. The MALLARD program^60^ was used to identify anomalous 16S rRNA gene sequences within multiple sequence alignments. The *nifH* sequences were translated and the amino acid sequences were aligned. Multiple sequence alignments were performed using CLUSTAL W^61^ in the current version of the BIOEDIT program^62^. The phylogeny of the deduced nitrogenase amino acid sequences or 16S rRNA gene sequences was analysed with MEGA software v 6.06^63^. Trees were constructed using neighbour-joining (NJ) algorithms^64^. Bootstrap analysis of 1000 replicates was performed for each consensus tree^65^. All clone sequences were submitted to GenBank under accession numbers JQ514111-JQ514149 and KF544966 for *nifH* sequences and JQ612137-JQ612154 and KF544967- KF544980 for 16S rRNA sequences.

### Statistical analysis

ANOVA with Tukey post hoc testing, independent sample t-tests, and paired sample t-tests were used to compare group differences. The SigmaStat program V2.03 was used for all statistical procedures.

## Additional Information

**How to cite this article**: Berrendero, E. *et al*. Nitrogen fixation in a non-heterocystous cyanobacterial mat from a mountain river. *Sci. Rep.* **6**, 30920; doi: 10.1038/srep30920 (2016).

## Figures and Tables

**Table 1:** Physical and chemical characteristics of water from the River Muga.

Variable (unit)	Mean (standard deviation)
Temperature (°C)	16.9 (1.6)
pH	8.3 (0.4)
O_2_ (mg l^−1^)	9.7 (0.5)
O_2_ (%)	103.7 (2.8)
Ca^2*^ (mg l^−1^)	183.8 (3.0)
Alkalinity (mg l^−1^ CaCO_3_)	169.7 (2.5)
NO_3_-N (mg l^−1^)	0.09 (0.02)
NH_4_-N (mg l^−1^)	0.02 (0.01)
FRP (μg l^−1^)	8.4 (2.9)
TFP (μg l^−1^)	18 (1.2)

Data are summarised as means (standard deviation) of five triplicate measurements at different times and days in May 2009 (FRP, filterable reactive P; TFP, total filterable P).

**Table 2:** N_2_ fixation, inorganic C (^13^C) and N (^15^NO_3_^−^ and ^15^NH_4_^+^) incorporation, biomass, and elemental composition in cyanobacterial communities from the Muga River.

Parameter	*Schizothrix* mat	*Rivularia* colonies
N_2_ fixation (nmol C_2_H_4_. mg chl^−1^.h^−1^)	40.3 (11.3)	66.4 (22.8)
N_2_ fixation (μg N.mg chl^−1^.h^−1^)*	0.38 (0.1)	0.62 (0.3)
Photosynthesis (μg C.mg chl^−1^.h^−1^)	149.6 (24.0)	449.7 (106.0)
N-NO_3_^−^ uptake (μg N.mg chl^−1^.h^−1^)	2.58 (3.1)	10.18 (2.6)
N-NH_4_^+^ uptake (μg N.mg chl^−1^.h^−1^)	1.65 (0.2)	4.04 (0.7)
Ash-free dw/dw	0.16 (0.1)	0.22 (0.04)
Water content (%)	54.0 (3.1)	34.15 (3.6)
Chlorophyll (μg.mg dw^−1^)	0.13 (0.05)	0.34 (0.01)
Carbon (mg.g dw^−1^)	93.6 (2.5)	299.0 (18.0)
Nitrogen (mg.g dw^−1^)	4.0 (1.1)	34.0 (6.0)
C/N	23.4	8.8

Data are the means (and standard deviations) of 3 to 10 determinations.

^*^Calculated using the theoretical molar ratio N_2_/C_2_H_2_ 3:1^19^
